# Supplementary material for: Response of Macrophyte Diversity in Coastal Lakes to Watershed Land Use and Salinity Gradient
Source: Int J Environ Res Public Health. 2022 Dec 10;19(24):16620. doi: 10.3390/ijerph192416620 (PMC9779085; doi:10.3390/ijerph192416620)
Supplement: Supplementary file 1 [file ijerph-19-16620-s001.zip › Table S1.pdf]

Table S1. Overview of geographical locations and morphological features of the studied coastal lakes. Morphometrical data source is reference 64. The catchment data listed in the table come from GIS-based measurements by authors.

Lake/Feature	**Ptasi Raj**	**Resko Przymorskie**	**Łebsko**	**Gardno**	**Kopań**	**Liwia Łuża**	**Wicko Przymorskie**	**Sarbsko**	**Jamno**	**Dołgie Wielkie**
Saline type of lakes	Brackish	Transitional				Freshwater				
Geographic coordinates	54°22′ N 18°48′ E	54°09′ N. 15°21′ E	54°43′ N. 17°25′ E	54°39′ N. 17°07′ E	54°29′ N. 16°27′ E	54°05′ N. 15°05′ E	54°33′ N. 16°38′ E	54°46′ N 18°38′ E	54°17′ N. 16°08′ E	54°42′ N. 17°12′ E
Water table area (ha)	53	577	7040	2338	786	211	1058	651	2196	136
Mean depth (m)	2.6	2.5	4.7	2.2	3.9	0.9	6.1	1.2	3.8	2.7
Volume (hm^3)	0.7	7.7	113.5	31.3	11.7	1.9	28.5	7.8	38.3	1.9
**Catchment area (ha):**										
**Total**	410.1	312 51.4	156 887.4	92 981.7	3 701.8	18 465.5	10 928.8	19 359.7	47 260.5	617.5
Lake area/ Total catchment area (%)	12.9	1.8	4.5	2.5	21.2	1.1	9.7	3.4	4.6	22.0
**Direct**	410.1	1257.7	12529.9	4220.8	2220.2	517.1	2031.5	1296.2	3105.6	617.5
Lake area/ Direct catchment area (%)	12.9	45.9	56.2	55.4	35.4	40.8	52.1	50.2	70.7	22.0
Direct catchment area /Total catchment area (%)	100	4	8	5	60	3	19	7	7	100
Hydrological connectivity	Permanent intrusion of seawater through Wisła Śmiała River channel and/or directly from Gdańsk Bay	Periodical seawater intrusion by a natural canal of Błotnica River	Periodical seawater intrusion by a natural canal of Łeba River	Periodical seawater intrusion by a natural canal of Łupawa River	Periodical seawater intrusion by a canal	Naturally isolated from the sea. No hydrological connectivity	Naturally isolated from the sea. No hydrological connectivity	Naturally isolated from the sea. No hydrological connectivity	Isolated from sea by a floodgate	Naturally isolated from the sea. No hydrological connectivity
