# Supplementary material for: Response of Macrophyte Diversity in Coastal Lakes to Watershed Land Use and Salinity Gradient
Source: Int J Environ Res Public Health. 2022 Dec 10;19(24):16620. doi: 10.3390/ijerph192416620 (PMC9779085; doi:10.3390/ijerph192416620)
Supplement: Supplementary file 1 [file ijerph-19-16620-s001.zip › Table S2.pdf]

Table S2. Physico-chemical properties of water in the studied costal lakes

		Baltic coastal lakes										
		**Dołgie Wielkie**	**Liwia Łuża**	**Sarbsko**	**Wicko Przym**	**Jamno**	**Gardno**	**Kopań**	**Łebsko**	**Resko Przym**	**Ptasi Raj**	**Total**
Parameter	**Type**	**F**	**F**	**F**	**F**	**F**	**T**	**T**	**T**	**T**	**B**	
Secchi Disk (Transparency)	Mean	19.60	29.83	33.43	31.36	28.18	38.80	27.67	32.92	34.73	77.75	33.76
	N	30	30	30	42	66	30	30	36	30	24	348
	St.D.	4.74	10.95	14.53	7.16	8.26	14.84	11.58	6.91	10.73	31.53	17.97
	CI -95 %	17.83	25.75	28.01	29.13	26.15	33.26	23.34	30.58	30.72	64.44	31.87
	CI +95 %	21.37	33.92	38.86	33.59	30.21	44.34	31.99	35.25	38.74	91.06	35.66
Temperature	Mean	16.31	17.84	15.72	16.62	16.26	15.66	16.97	15.82	16.04	16.03	16.33
	N	30	30	30	42	66	30	30	36	30	24	348
	St.D.	4.94	4.04	3.94	4.55	3.98	3.75	4.60	4.55	3.21	5.46	4.28
	CI -95 %	14.47	16.33	14.25	15.21	15.28	14.26	15.25	14.28	14.84	13.73	15.88
	CI +95 %	18.15	19.35	17.19	18.04	17.24	17.06	18.69	17.36	17.24	18.34	16.78
pH	Mean	8.78	8.75	8.52	8.66	8.80	8.68	8.97	8.46	8.83	7.89	8.66
	N	30	30	30	42	66	30	30	36	30	24	348
	St.D.	0.48	0.38	0.36	0.29	0.37	0.33	0.47	0.23	0.29	0.31	0.43
	CI -95 %	8.60	8.61	8.39	8.57	8.71	8.55	8.80	8.39	8.71	7.75	8.62
	CI +95 %	8.95	8.89	8.66	8.75	8.89	8.80	9.15	8.54	8.94	8.02	8.71
DO%	Mean	103.44	124.47	97.95	104.32	106.30	99.61	116.97	87.76	101.40	86.78	103.32
	N	30	30	30	42	66	30	30	36	30	24	348
	St.D.	19.69	26.25	27.00	20.51	16.58	19.13	19.59	17.95	25.60	37.55	24.50
	CI -95 %	96.09	114.66	87.87	97.93	102.22	92.46	109.66	81.69	91.84	70.93	100.73
	CI +95 %	110.79	134.27	108.03	110.71	110.37	106.75	124.29	93.84	110.96	102.64	105.90
Salinity	Mean	0.05	0.40	0.04	0.13	0.13	1.57	1.09	2.73	2.48	6.89	1.28
	N	30	30	30	42	66	30	30	36	30	24	348
	St.D.	0.01	0.24	0.01	0.06	0.06	1.83	0.30	0.73	1.28	0.71	1.95
	CI -95 %	0.04	0.31	0.03	0.11	0.12	0.89	0.97	2.48	2.00	6.59	1.08
	CI +95 %	0.05	0.49	0.04	0.15	0.15	2.25	1.20	2.98	2.95	7.19	1.49

TOC	Mean	38.37	22.48	15.13	14.31	20.30	9.90	27.68	15.50	12.58	8.78	18.66
	N	30	30	30	42	66	30	30	36	30	24	348
	St.D.	14.08	13.31	6.88	4.02	7.05	3.57	15.08	4.36	4.53	3.95	11.60
	CI -95 %	33.11	17.51	12.56	13.06	18.57	8.57	22.05	14.03	10.89	7.12	17.44
	CI +95 %	43.63	27.45	17.70	15.57	22.03	11.23	33.31	16.98	14.27	10.45	19.88
DOC	Mean	18.12	14.74	8.68	10.28	10.23	6.09	13.82	8.34	7.23	5.37	10.34
	N	30	30	30	42	66	30	30	36	30	24	348
	St.D.	3.96	6.56	3.89	2.95	3.89	2.95	6.95	3.56	2.79	2.38	5.46
	CI -95 %	16.64	12.29	7.23	9.36	9.28	4.99	11.23	7.14	6.19	4.37	9.76
	CI +95 %	19.60	17.19	10.14	11.20	11.19	7.19	16.42	9.55	8.27	6.37	10.91
$N-NO_3$	Mean	0.34	0.58	0.61	0.66	0.84	0.85	0.85	0.73	0.98	1.03	0.75
	N	30	30	30	42	66	30	30	36	30	24	348
	St.D.	0.17	0.48	0.36	0.52	0.91	0.56	0.66	0.54	0.92	0.71	0.67
	CI -95 %	0.28	0.40	0.48	0.50	0.62	0.64	0.60	0.54	0.64	0.73	0.68
	CI +95 %	0.40	0.76	0.75	0.82	1.07	1.06	1.10	0.91	1.32	1.33	0.82
$N-NH_4$	Mean	0.24	0.57	0.16	0.18	0.40	0.43	0.13	0.35	0.35	0.38	0.32
	N	30	30	30	42	66	30	30	36	30	24	348
	St.D.	0.13	0.43	0.09	0.14	0.33	0.36	0.09	0.23	0.44	0.28	0.31
	CI -95 %	0.19	0.41	0.13	0.13	0.32	0.30	0.10	0.27	0.19	0.26	0.29
	CI +95 %	0.29	0.73	0.20	0.22	0.48	0.57	0.16	0.43	0.52	0.50	0.35
$P-PO_4$	Mean	0.10	0.11	0.11	0.18	0.55	0.05	0.15	0.04	0.15	0.30	0.21
	N	30	30	30	42	66	30	30	36	30	24	348
	St.D.	0.04	0.09	0.12	0.08	0.29	0.03	0.07	0.02	0.10	0.14	0.23
	CI -95 %	0.08	0.07	0.06	0.15	0.48	0.04	0.12	0.04	0.11	0.24	0.18
	CI +95 %	0.11	0.14	0.15	0.20	0.62	0.06	0.17	0.05	0.18	0.36	0.23
TP	Mean	0.31	0.49	0.33	0.48	0.87	0.16	0.35	0.28	0.40	0.57	0.47
	N	30	30	30	42	66	30	30	36	30	24	348
	St.D.	0.11	0.38	0.30	0.25	0.60	0.08	0.18	0.14	0.21	0.29	0.40
	CI -95 %	0.27	0.35	0.21	0.40	0.72	0.13	0.28	0.23	0.32	0.44	0.42
	CI +95 %	0.35	0.64	0.44	0.56	1.02	0.19	0.42	0.33	0.48	0.69	0.51
