# Supplementary material for: Response of Macrophyte Diversity in Coastal Lakes to Watershed Land Use and Salinity Gradient
Source: Int J Environ Res Public Health. 2022 Dec 10;19(24):16620. doi: 10.3390/ijerph192416620 (PMC9779085; doi:10.3390/ijerph192416620)
Supplement: Supplementary file 1 [file ijerph-19-16620-s001.zip › Table S3.pdf]

Table S3. List of macrophyte communities of coastal lakes in the Baltic Sea. Asterix denotes marine species

**Ecological groups**		**Community**	**Type of coastal lakes**									
			**Brackish**	**Transitional lakes**				**Freshwater lakes**				
			Ptasi Raj	Resko Przymorskie	Łebsko	Gardno	Kopań	Liwia Łuża	Wicko Przymorskie	Sarbsko	Jamno	Dołgie Wielkie
Submerged	Charophytes	*Nitellopsis obtusae*	5*		1							
		*Chara canescens**	5									
		Chara fragilis	2									
		Chara contraria	2									
		Chara aspera				1						
	Elodeides	*Myriophyllum spicatum*	3	3	1	3	7		3	5	2	
		Potamogeton pectinatus	7	3		1				3	2	
		Potamogeton perfoliatus		3	1	2			2	5	2	
		Ceratophyllum demersum	2			1		1				
		Najas marina			3							
		Potamogeton lucens			1			1	1			
		Potamogeton crispus		1							1	
		Elodea canadensis				1						
		*Utricularia vulgaris**					1					
	Nymphaeides	*Nuphar lutea*		2	3	3		5	2	1		5
		Nymphaea sp.			2				1			7
		Polygonum amphibium f.natans			2						1	2

		Potamogeton natans							1			2
		Hydrocharis morsus-ranae					1				2	
		Stratiotes aloides									2	
		Sagittaria sagittifolia		1								
Emerged	Helophytes	*Phragmites australis*	7	7	9	9	8	9	9	7	9	5
		Scirpus lacustris		3	2	5	2		3	5	3	7
		Typha angustifolia		8	5	3	5		5	2	3	3
		Glyceria maxima		2	3	5				2	3	7
		Carex acutiformis		2		2	3	5			2	
		Phalaris arundinacea		3	2	2		3		2	2	3
		*Scirpus tabernaemontani**		1	3		2		1	2	1	
		Typha latifolia		1	5	3		2		1	2	1
		Sparganium erectum		2	2	2	1	2	1	3	2	
		Butomus umbellatus		2	3	3		1		2	2	
		Iris pseudacorus		2	1	2	1	2	2		1	1
		*Scirpus maritimus**		2	3	2	2	1		1	1	
		Eleocharis palustris			1				1	3		5
		Carex paniculata						1		1		3
		Acorus calamus			2	2				2		
		Carex rostrata										5
		Thelypteris palustris						3				
		Cicuta virosa			2			2				
		Equisetum fluviatile										2
		Rumex hydrolapathum								1		

		Comarum palustre										2
		Rorippa amphibia									1	
		Carex acuta			3							
		Carex riparia										2
**Macrophyte characteristics**												
No of communities (S)			8	18	23	19	10	13	13	18	20	17
Biodiversity, Shannon-Wiener index (H')			1.42	0.91	1.64	1.13	1.12	1.07	0.73	2.89	0.47	2.10
Evenness, Pielou Index (J')			0.68	0.31	0.52	0.38	0.49	0.42	0.29	0.60	0.16	0.60
Vegetation coverage (C_{max})			2.5	0.8	1.4	1.2	1.5	1.0	1.5	1.5	1.2	1.5
Share of the phytolittoral area in the total lake area (N,%)			62.5	5.1	23.8	13.7	35.5	13.7	19.9	44.4	13.2	30.4
Colonisation index (Z)			0.70	0.05	0.27	0.14	0.36	0.14	0.32	0.46	0.18	0.36
Share of Charophytes (%)			23.3	0.0	0.0	<0.1	0.0	0.0	0.0	0.0	0.0	0.0
Share of Nympheides (%)			0.0	0.3	4.3	1.7	<0.1	12.0	0.6	0.1	0.2	26.1
Share of Emerged (%)			47.5	95.2	88.2	96.5	70.0	87.9	96.1	55.8	98.5	73.9
Share of Submerged (%)			52.5	4.8	1.8	3.5	30.0	12.1	3.9	44.2	1.5	26.1
Submerged / Emerged (%)			1.1	19.8	49.0	<0.1	2.3	7.3	24.6	1.3	<0.1	0.4
Share of marine communities (%)			11.7	0.5	3.0	0.7	0.1	0.5	0.0	0.1	0.3	0.0

*Percentage area coverage: 9>75%; 8=50-75%; 7=25-50%; 6=10-25%; 5=5-10%; 4=2.5-5%; 3=1-2.5%; 2=0.1-1%; 1=<0.1%
